# Protein Quality Changes of Vegan Day Menus with Different Plant Protein Source Compositions

**DOI:** 10.3390/nu14051088

**Published:** 2022-03-04

**Authors:** Zaray Rojas Conzuelo, Natalie S. Bez, Steffen Theobald, Katrin A. Kopf-Bolanz

**Affiliations:** School of Agricultural, Forest, and Food Sciences, Bern University of Applied Sciences, 3050 Zollikofen, Switzerland; zaray.rojasconzuelo@bfh.students.ch (Z.R.C.); natalie.bez@bfh.ch (N.S.B.); steffen.theobald@bfh.ch (S.T.)

**Keywords:** protein quality, plant protein, vegan diets

## Abstract

To underline the importance of protein quality in plant-based diets, we estimated the protein quality of different exclusively plant-protein-based day menus that are based on the “planetary health diet” developed by the EAT-Lancet Commission. PDCAAS and DIAAS were used to estimate the protein quality (PQ) and fulfilling of the amino acid recommendation for adults in vegan daily menus based on the planetary health diet: 2 days with only low-quality (LQ) protein sources and 2 days with low + high-quality (HQ) protein sources. The protein quality of Day 1LQ (DIAAS 76, PDCAAS 88) was increased by the addition of high-quality protein sources (HQPS): Day 1HQ (DIAAS 94, PDCAAS 98). Day 2LQ had a low PQ (DIAAS 71, PDCAAS 74), but when HQPS were used (Day 2HQ), the PQ increased (DIAAS 83, PDCAAS 88). Scenarios (day 1HQ, day 1LQ, and day 2 HQ) were classified as of good PQ. However, day 1LQ had a low protein quality. Consuming HQPS in a vegan diet can help to fulfil the recommendation of essential amino acids. This work served to understand and apply methods to estimate protein quality that can be applied to optimize protein mixtures to fulfil amino acid requirements in the future.

## 1. Introduction

A plant-based diet with a minimum number of animal-sourced foods provides health and environmental benefits. However, there might be a lack in the absorption of certain indispensable amino acids that are important for health. Even though plant foods contain all the nine indispensable amino acids (IAA), the IAA profile may not be optimal according to the established requirements for human needs [1,2]. Additionally, the so-called antinutrients may hinder the digestibility of plant-based nutrients [3]. Protein quality reflects to what extent a food protein source or a diet fulfils the metabolic demand for amino acids and nitrogen, and thus, whether the protein is used efficiently by the body [4]. To estimate protein quality, two factors are considered: the indispensable amino acid content and digestibility. Digestibility is an attribute that also depends on the individual’s metabolism, but for protein quality estimation purposes, it will be referred to only as the amount of amino acid or protein absorbed by the digestive tract [4,5]. 

There are various methods to evaluate protein quality. The Food and Agricultural Organization of the United Nations (FAO) currently recommends the method of amino acid scoring, for which the Protein Digestibility-Corrected Amino Acid Score (PDCAAS) has been widely used since 1989, when it was first proposed. It uses a reference protein that is thought to meet all indispensable amino acid requirements and compares it with the test protein for a specific age group. It aims to reflect the overall efficiency of protein utilization in terms of digestibility (absorbed proportion of the food protein) and indispensable amino acid profile [6]. However, it is recognized that the PDCAAS has some limitations. Therefore, the “Digestible Indispensable Amino Acid Score” (DIAAS) was proposed in 2011 as an improved method [7]. One of the most important new features of the DIAAS method is that it uses a score based on digestibility of individual dietary indispensable amino acids instead of the crude protein. Moreover, it is measured at the end of the small intestine (ileal digestibility), which is a more accurate representation of amino acid digestion and absorption than fecal digestibility in PDCAAS. Nevertheless, if data for amino acid digestibility are not available, values of crude protein digestibility are accepted as an equivalent. The value of protein quality is not truncated, so it is possible to obtain a score above 100 to express extra health benefits unless calculated for a mixed diet or a sole source of food [7].

The amino acid adequacy of plant-based diets was discussed in a recent review of Mariotti et al., 2019 [8]. They concluded that traditional vegetarian diets provide sufficient protein and amino acids when the sources are at least minimally varied, but still, a small percentage of vegans may experience insufficient consumption. However, digestibility is generally not contemplated in that study, and it is often argued that the bioavailability of animal-sourced proteins is not similarly to those of plant proteins but might be comparable to the digestibility of plant protein isolates [8].

In 2019, the EAT-Lancet Commission on Food, Planet and Health proposed a diet (the EAT-Lancet diet) that is both healthy and within planetary boundaries. The diet follows a flexitarian approach, which is constituted predominantly by a diversity of plant-based foods, but it can be adapted to the full spectrum of plant-based diets. It emphasizes the consumption of vegetables, fruit, legumes, whole grains, nuts, and fish and limits red meat and starchy vegetables, while the intake of eggs, poultry, and dairy foods is optional. As only ranges of food groups are given, this is not a rigorous diet nor an exact prescription. It should be adapted to the individual energy requirements, culture, location, demography, and food preferences [9].

This study aimed to evaluate the changes in dietary protein quality when only low-quality protein is consumed and how the addition of high-quality protein sources influences when consuming exclusively plant-based foods (vegan diet). It is based on a case study in the frame of the EAT-Lancet diet. We estimate the protein quality of two menu days in two scenarios: (1) only “low-quality” protein sources, (2) “low” + “high-quality” protein sources.

## 2. Materials and Methods

To investigate the protein quality of a vegan diet, certain vegan menus of the EAT-diet were used as examples. Since the EAT-diet should be adapted to the individual and local context, the menus were designed for an individual case of a healthy Swiss male with 78 kg body weight, 30 years of age, moderate level of physical activity, and an average energy requirement of 2500 kcal/day, who consumes a healthy vegan diet. The daily protein requirement was 64.8 g/day, based on an adequate protein intake for adults of 0.83 g/kg body weight per day [10].

### 2.1. Menu Creation

First, a list of plant protein sources from different food groups with their respective DIAAS value was created. The list contained only foods available in the Swiss Food Composition Database to be in line with the case study. The list of food items was dichotomized according to the following criteria for judged quality [7]: DIAAS < 75 was defined as low-quality protein sources (LQPS), DIAAS ≥ 75 as high-quality protein sources (HQPS).

Four different daily menus were made. First, two where only LQPS were used, including five meals: breakfast, lunch, dinner, and two snacks. The portions were set according to the recommendation by the current Swiss Nutrition Policy. The final quantities per day of each food group were set according to the ranges proposed in the EAT-diet and the daily protein requirement (64.8 g).

Afterwards, based on the two menus, a scenario with high-quality protein was made. For this, some of the LQPS were substituted by protein sources with higher DIAAS (Table 1). Since not many high-quality protein sources were available, foods with higher DIAAS were used to substitute LQPS (not necessarily items with DIAAS > 75). The quantities of food were adjusted to obtain 64.8 g of protein per day for each menu.

On Day 1 LQ, three LQPS foods were substituted with three HQPS. On day 2, four items were substituted in the same manner (Table 1).

In the menus, the following food items were not considered: water and other liquids; minor ingredients such as sauces, herbs, spices, added oils, and added sugars due to their low or null protein content.

### 2.2. Calculation of Protein Quality

First, the quantities of dry grains and dry pulses were converted to cooked weight (they were initially in grams of dry grain for the calculation of food ranges according to EAT diet). For black beans, white rice, and brown rice, a ratio of 3:1 cooked to dry was used. For chickpeas, oats, lentils, and quinoa, a ratio of 2:1 cooked to dry was used.

Information about protein and IAA content, true protein digestibility (TPD), and Ileal IAA digestibility were gathered for each food item (Tables S1 and S2) considering the final state of consumption (e.g., raw, cooked, roasted). Second, the protein quality was calculated in an Excel spreadsheet with the methods PDCAAS and DIAAS for mixed protein sources as described by FAO/WHO/UN (2007) and FAO (2013), respectively, according to the requirements established for adults (Table 2). The results of PQ ≥ 100 were classified as excellent PQ, of 0.75–0.99 as good, and PQ < 0.75 as low, respectively.

$$PDCAAS=\frac{mg\;of\;limiting\;amino\;acid\;in\;1\;g\;of\;test\;protein}{mg\;of\;the\;same\;amino\;acid\;in\;1\;g\;of\;reference\;protein\;}\times True\;fecal\;digestibility$$

$$DIAAS=Lowest\;value\;\left(\frac{mg\;of\;digestible\;dietary\;indispensable\;amino\;acid\;in\;1\;g\;of\;dietary\;protein}{mg\;of\;the\;same\;dietary\;indispensable\;amino\;acid\;in\;1\;g\;of\;the\;reference\;protein}\right)$$

## 3. Results

### 3.1. Food Group Ranges

The menus were adapted to provide a protein quantity of a minimum of 64.8 g each (Table 3). Due to the need to comply with the ranges of the EAT diet, the amount of protein per day varied between 64.9 g and 65.3 g(Table 4). Because LQ protein sources were substituted with HQ proteins sources, and some sources contain more protein than others, some of the food ranges differ between days (Table 5). For instance, for day 1 LQ 120 g of legumes provide 23 g of protein; whereas for the same day but in the scenario with high quality, 78 g of legumes were sufficient to provide 27 g. The same case is for day 2, where the quantity of legumes for the LQ scenario is higher (96.5 g) but contributes to a lower amount of protein (16.61 g) compared to HQ, where 72.5 g legumes provide 22 g of protein (Table 4 and Table 5).

### 3.2. Amino Acid Score of the Daily Menus

**Figure 1:**
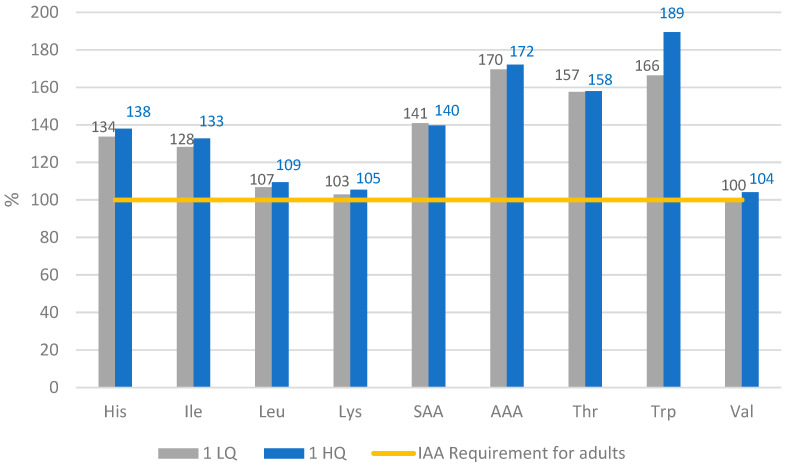
Amino Acid Score of day 1. His = histidine, Ile = isoleucine, Leu = leucine, Lys = lysine, SAA = Sulphur amino acids, AAA = Aromatic amino acids, Thr = threonine, Trp = tryptophan, Val = valine, IIA = Indispensable Amino Acids. Requirement based on method DIAAS.

**Figure 2:**
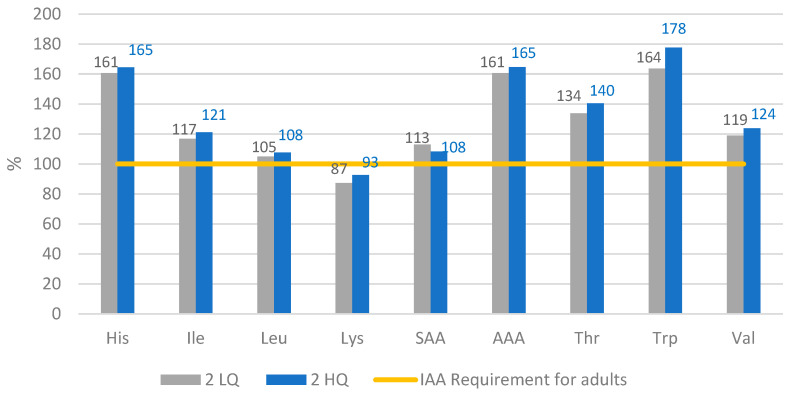
Amino Acid Score of day 2. His = histidine, Ile = isoleucine, Leu = leucine, Lys = lysine, SAA = Sulphur amino acids, AAA = Aromatic amino acids, Thr = threonine, Trp = tryptophan, Val = valine, IIA = Indispensable Amino Acids. Requirement based on method DIAAS.

Figure 1 and Figure 2 show the Amino Acid Score (AAS), which indicates to what extent the amino acid content fulfils the requirements established by PDCAAS and DIAAS on a percentage scale. For day 1 in both scenarios, the AA pattern was fulfilled according to the reference pattern (Table 2). For lysine, valine, threonine, leucine, isoleucine, and histidine, the value is not fulfilled with legumes and cereals alone, but the vegetables and fruits complete the requirements (data not shown). Lysine, valine, and leucine values are more likely to not reach the recommendation. In contrast, the aromatic amino acids (AAA), tryptophan and trypsin, display a surplus.

On day 2, the menu provides does not provide the quantity of lysine established as a requirement. Even though the quantity in grams of cereals for day 2 is virtually the same as on day 1, the protein contribution is greater. This can explain the deficit of lysine for day 2, since cereals are generally more deficient in lysine [19]. The rest of IAA comply and/or exceed the reference, especially AAA for all the menus.

### 3.3. Protein Quality

#### 3.3.1. Protein Digestibility-Corrected Amino Acid Score (PDCAAS)

Lysine was the limiting AA in three out of four scenarios. Only in D1LQ, valine was most limiting but closely followed by lysine. The rest of the IAAs in D1HQ, D2LQ, and D2HQ fulfilled or exceeded the recommended intake for adults. 

**Figure 3:**
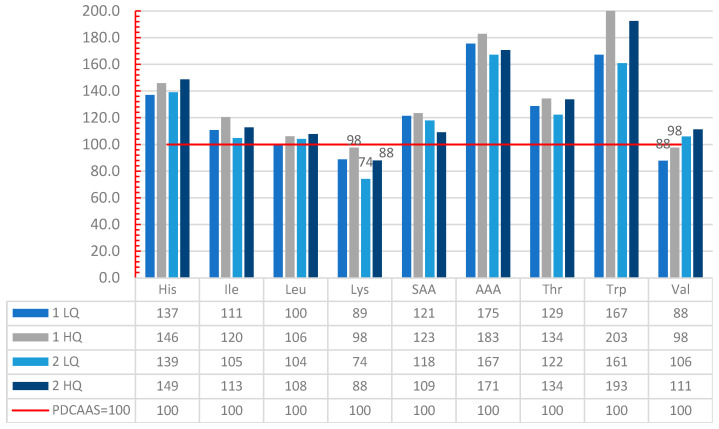
Amino Acid Reference ratio for the four day menus with the method PDCAAS. His = histidine, Ile = isoleucine, Leu-= leucine, Lys = lysine, SAA = Sulphur amino acids, AAA = Aromatic amino acids, Thr = threonine, Trp = tryptophan, Val = valine.

Only D2LQ had a low quality of protein, even though this value (PDCAAS 74) is very close to the cut-off value of 75. The rest of the day menus had PDCAAS higher than 75 (D1LQ = 88, D1HQ = 98, D2HQ = 88); thus, we considered them of good quality (Figure 3). 

Interestingly, a scenario with only low-quality protein sources (D1LQ) had the same PDCAAS value of 88 as one with added high-quality protein (2HQ).

#### 3.3.2. Digestible Indispensable Amino Acid Score (DIAAS)

**Figure 4:**
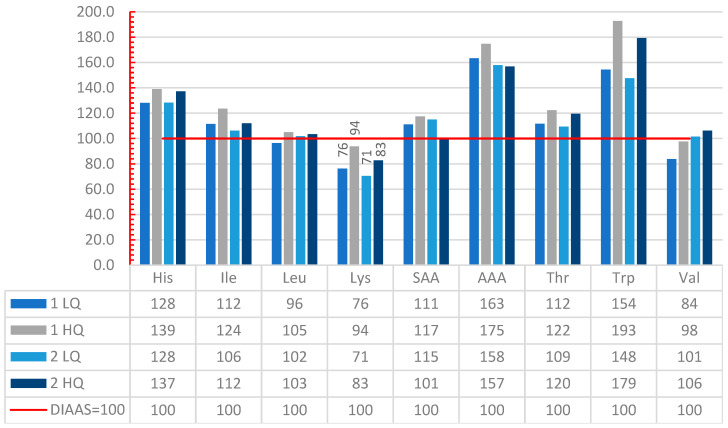
Amino Acid Reference ratio for the four day menus with the method DIAAS. His = histidine, Ile = isoleucine, Leu = leucine, Lys = lysine, SAA = Sulphur amino acids, AAA = Aromatic amino acids, Thr = threonine, Trp = tryptophan, Val = valine.

With this method, the most limiting amino acid was lysine in all the scenarios. Both scenarios with low-quality proteins had a lower DIAAS: 0.76 and 0.71 for 1LQ and 2 LQ, respectively. For day 2 in LQ and HQ, only lysine is limiting, and the rest of the IAA exceed the reference, whereas for day 1, valine and leucine are also limiting (Figure 4).

Regarding judged quality, day 1 (HQ and LQ) is considered to provide good quality in both scenarios and with both methods. Day 1 HQ has the highest protein quality: 94 with DIAAS and 98 with PDCAAS (Table 6 and Table 7). The only scenario with low-quality protein was on day 2 LQ. When HQPS are added in this scenario, the protein quality increases by 17% (DIAAS) and 19% (PDCAAS) and the judged protein quality is good.

## 4. Discussion

Protein is an essential component of a healthy diet that allows the correct growth and maintenance of the 25,000 proteins in the human genome. The amount required to fulfil the latter functions is defined as the dietary protein requirement [4]. Such requirement depends on the metabolic demands of the organism (age, physical activity expenditure, and energy expenditure), as well as the efficiency of utilization of the protein source. Hence, dietary requirement = metabolic demand/efficiency of utilization. 

The recommended daily allowance (RDA) to meet the requirements of protein of 97.5% of the healthy adults is 0.8 g/kg body weight per day [4]. As expected, in this study, the values of PDCAAS were higher than of DIAAS. The reason is that DIAAS values are calculated (when data are available) with ileal digestibility, which is more accurate than fecal digestibility used for PDCAAS, so PDCAAS may overestimate the protein quality. With both methods, 1 HQ had the highest PQ, very close to 100. This indicates that the IAA quantity is very close to that of the requirements. 

With both methods, the PQ of the HQ scenarios were classified as good, and with both methods, the PQ of the day 2 LQ was low. In this regard, the results are consistent. However, the protein quality of day 1 LQ was very different with PDCAAS (88) vs DIAAS (76) (Table 6 and Table 7). Between methods, the other scenarios had a smaller variation. Table 1 shows that on day 1, only three food items were substituted (soy drink for almond drink, tofu for beans, and lentils for chickpeas), and this was sufficient to improve the quality by 11% (PDCAAS) and 24% (DIAAS) (Table 7). The most limiting amino acid in all the scenarios was lysine. Day 2 LQ and HQ were already deficient in this nutrient before considering digestibility. The fact that one scenario with LQPS had good PQ and the other low PQ, can be partly explained by synergies between the different protein sources. The substitution of LQPS with HQPS in this study was made on a gram of protein contribution basis so that the protein quality is comparable. For instance, to obtain the same quantity of protein (9 g), 200 g of cooked chickpeas were substituted with 120 g of cooked lentils because the protein content of lentils is higher. However, when substituting a protein source in practice, it is more likely that the substitution would be one to one, which would result in a higher total protein per day. The resulting protein quality would probably be higher as well since the proportion of HQPS would be greater. In the same manner, it could lead to changes in the intake of energy and other macronutrients. Further research could analyze the impact of substituting LQPS with HQPS on other macronutrients. For instance, replacing 120 g of cooked pasta with 225 g of cooked brown rice (as in Day 2), would result in an increase of carbohydrates (from 35.2 g to 52.9 g, data not shown) and calories (from 174 cal to 252 cal, data not shown) [20]. 

There were some limitations in this study. The main challenge was the availability of data on digestibility, especially for fruits and vegetables. Several foods belonging to those groups were assumed to have the same value, e.g., raw carrot and raw apple for both ileal digestibility of IAA and fecal digestibility of crude protein. Furthermore, FAO (2013) recommends measuring digestibility in humans (for DIAAS), but information on these characteristics is scarce [7]. When data are not available, it is possible to use studies performed in growing pigs or rats. For PDCAAS, the recommended assay is in rats. As not all the food items in this study have digestibility studies in vivo, some data were taken from in vitro studies. However, some studies suggest that in vitro assays provide an accurate estimate of the TPD [21,22,23]. For the estimation of DIAAS, there is no information on individual IAA digestibility for every food item. For these cases, the value of the crude protein digestibility is used instead, as recommended by the FAO (2013) [7]. 

Moreover, the calculation relies on the quality of each experimental value for IAA content, IAA ileal digestibility, and crude protein digestibility. Thus, the results might carry considerable compounding errors. Other aspects, such as lack of specific nitrogen-to-protein conversion factors to calculate protein content or not considering the food matrix [24], remain a limitation, since not enough data to address them are available. According to Craddock et al. [24], PDCAAS is currently the most appropriate approach for use in Western adults who follow a plant-based diet. For that reason, this method was also used, and the outcomes were compared to DIAAS. The two methodologies showed the same trend, and the final classification (low or high protein quality) was the same. In this work, only 2-day menus were evaluated. However, a 7-day menu would be more robust since all weekdays (including weekends) are represented. Additionally, it was assumed that by following the ranges of the EAT report, the nutrient and energy adequacy is fulfilled, so it was not addressed here. 

The menus were designed following the ranges proposed by the EAT-lancet Commission for a healthy diet, so unhealthy food or meat/dairy analogues are not considered. The plates of the menus are all based on whole foods that need a considerable amount of time and skill to be prepared. The question of how realistic and affordable this pattern is, is not addressed in the present study. We only analyzed two days, and a longer diet might be more representative. However, it shows clear tendencies and can be compared to a real diet analysis of vegan people in the future.

One of the strengths of this study is that home processing was considered. The different cooking techniques, such as soaking followed by boiling, generally increase the digestibility of the proteins when compared to the raw food [25]. The reason is that processing inactivates or reduces the amount of some of the compounds that limit the digestion, namely antinutritional factors and that are present in plant protein sources [3,26]. 

To our knowledge, this is the first study that estimates the DIAAS and PDCAAS of a vegan mixed diet considering also fruits and vegetables. Other studies have evaluated the PQ of mixed diets [27,28] but with a more limited database of food items, mainly accounting for grains and legumes and not considering fruits and vegetables. Overlooking those items could lead to underestimating the quality and quantity of protein, especially in plant-based diets [24,29]. Furthermore, our findings are in line with a recent study from Salome et al. (2020) that analyzed a representative French national dietary survey with 1341 participants and found that the plant proteins ingested were not very diverse and even less diverse for higher plant protein intake. They further concluded that it is important that the plant-protein origins are diverse and do not originate mostly from refined grains [30]. It might be also interesting to compare the environmental impact of using high protein quality sources instead of low protein quality sources in the future.

Especially for the elderly and people that have a problem reaching the recommended protein intake amounts, it is very important to consider protein sources with a high protein quality that might even result in beneficial physiological consequences, such as maintaining muscle mass [31]. 

In this case, the need for a higher protein intake in the diet, which may be difficult for elderly people or may also result in an increased calorie intake, can be avoided [31]. 

## 5. Conclusions

This study contributes to the understanding of protein quality in vegan dietary patterns and highlights the importance of incorporating high-quality protein sources in diets that rely exclusively on plant-based foods. It further shows how harmonized amino acid scorings can be used as tools to evaluate the protein quality of every-day diets. The planetary health diet in a vegan version can, indeed, provide a good protein quality. However, even when consuming plant protein from diverse sources and in the amounts recommended by the EAT-Lancet diet, the protein quality might be low when only low-quality protein sources are present in the diet. The substitution of some items with high-quality protein sources considerably increased the protein quality and turned it from a low-quality protein menu into a high-quality one. Therefore, it is important to pay attention to the quality of the food source, and not only to the recommended quantity. Furthermore, especially people that have problems reaching the protein recommendation per day should take care and include high-quality proteins or balancing protein combinations in their diet. It is important to develop guidelines or tools that dieticians can use to transfer these findings into practice. 

## Figures and Tables

**Table 1:** Protein source changes from LQ to HQ menus.

Day	LQPS Substituted by	HQPS
1	Almond drink. DIAAS 41 ^1^	Soy drink. DIAAS 117 ^2^
1	Chickpeas, cooked. DIAAS 67 ^9^	Lentils, cooked. DIAAS 75 ^5^
1	Black beans, cooked. DIAAS 63 ^3^	Tofu. DIAAS 97 ^2^
2	Chickpeas, cooked. DIAAS 67 ^9^	Yellow pea. DIAAS 67 ^7^
2	Wholewheat bread. DIAAS 20 ^8^	Quinoa, cooked. DIAAS 72 ^6^
2	Pasta, wholewheat. DIAAS 36 ^4^	Brown rice. DIAAS 42 ^8^
2	Peas, cooked. DIAAS 68 ^1^	Tofu. DIAAS 97 ^2^

LQPS = Low-quality protein sources, HQPS = High quality protein sources, ^1^ [11], adults ^2^ [12], adults ^3^ [13], children ^4^ [14], adults ^5^ [5], ^6^ [15] (PDCAAS used in absence of DIAAS), ^7^ [16], ^8^ [17], 0.5–3-year-old child ^9^ [18].

**Table 2:** IAA reference pattern (mg/g). Adapted from WHO/FAO/UN 2007 for PDCAAS and from FAO 2013 for DIAAS.

Age Group (Years)	His	Ile	Leu	Lys	SAA	AAA	Thr	Trp	Val
PDCAAS: Adults (>18 years)	15	30	59	45	22	38	23	6	39
DIAAS: Older child, adolescent, adult (>3 years)	16	30	61	48	23	41	25	6.6	40

PDCAAS = Protein Digestibility-Corrected Amino Acid Score, DIAAS = Digestible Indispensable Amino Acid Score, His = histidine, Ile = isoleucine, Leu = leucine, Lys = lysine, SAA = Sulphur. amino acid, AAA = Aromatic amino acid, Thr = threonine, Trp = tryptophan, Val = valine.

**Table 3:** Compositions of the menus for day 1 and day 2 in their versions with low-quality protein sources (LQ) and low + high-quality protein sources (HQ).

	Day 1 LQ	Day 1 HQ
Breakfast	**Apple cinnamon oats**	**Apple cinnamon oats**
	120 g cooked oats (60 g dry)	120 g cooked oats (60 g dry)
	10 g roasted pumpkin seeds	10 g roasted pumpkin seeds
	285 g almond drink 7% almond	100 g soy drink
	120 g raw apple	120 g raw apple
Snack 1	75 g Whole grain cracker (40 g dry grain)	75 g Whole grain cracker (40 g dry grain)
	60 g Bean spread	50 g tofu bites (10 g dry soybean)
Lunch	**Vegan pizza**	**Vegan pizza**
	100 g whole wheat bread (55 g dry grain)	100 g whole wheat bread (55 g dry grain)
	47.5 g tomato sauce	47.5 g tomato sauce
	55 g red onion	55 g red onion
	110 g peppers (green and yellow)	110 g peppers (green and yellow)
	37 g broccoli	37 g broccoli
	72 g cauliflower	72 g cauliflower
Snack 2	20 g roasted peanuts	20 g roasted peanuts
	120 g pear	120 g pear
Dinner	**Safran chickpea risotto**	**Safran lentil risotto**
	225 g cooked rice (75 g dry)	225 g cooked rice (75 g dry)
	200 g cooked chickpeas (100 g dry)	120 g cooked lentils (60 g dry)
	50 g cooked beetroot	50 g cooked beetroot
	50 g cooked carrot	50 g cooked carrot
	75 g green salad with Italian dressing	75 g green salad with Italian dressing
	**Day 2 LQ**	**Day 2 HQ**
Breakfast	**Bread with peanut butter and banana slices**	**Bread with peanut butter and banana slices**
	20 g peanut butter	20 g peanut butter
	100 g whole wheat bread (55 g dry grain)	100 g whole wheat bread (55 g dry grain)
	120 g banana, raw	120 g banana, raw
Snack 1	**Bread with hummus dip**	**Quinoa cracker with pea basil spread**
	60 g hummus (27 g chickpeas)	60 g yellow pea spread (15 g dry)
	100 g whole wheat pita bread	60 g quinoa cracker (30 g dry seed)
	120 g strawberries	120 g strawberries
Lunch	**Portobello tacos**	**Portobello tacos**
	100 g corn tortilla (60 g grain)	100 g corn tortilla (60 g grain)
	100 g cooked beans (33 g dry beans)	100 g cooked beans (33 g dry beans)
	100 g portobello mushroom	100 g portobello mushroom
	50 g onions, raw	50 g onions, raw
	100 g tomato, cooked	100 g tomato, cooked
	30 g red cabbage, raw	30 g red cabbage, raw
Snack 2	30 g corn chips (18 g dry grain)	30 g lentil chips
	50 g avocado, raw (guacamole)	50 g avocado, raw (guacamole)
Dinner	**Pea pasta salad**	**Marinated tofu with brown rice**
	120 g cooked pasta (45 g dry grain)	225 g cooked brown rice
	100 g cooked peas (50 g dry)	100 g tofu (20 g dry soybean)
	10 g sunflower seeds, roasted	10 g sunflower seeds
	120 g pepper, cooked	120 g pepper, cooked
	100 g green salad with French dressing	100 g green salad with French dressing

**Table 4:** Contribution of protein (g) per food group for each scenario.

	1 LQ	1 HQ	2 LQ	2 HQ
Fruits	0.8	0.8	3.1	3.1
Legumes	23.0	27.0	16.6	21.9
Nuts and seeds	10.9	6.6	6.5	6.5
Vegetables	6.8	6.8	7.6	7.6
Whole grains	23.8	23.8	31.4	26.1
Total per day	65.3	65.0	65.2	65.3

LQ = Low-quality day menu, HQ = High-quality day menu.

**Table 5:** Quantity of each food group per day.

Food Group	EAT-Reference Diet	1 LQ	1 HQ	2 LQ	2 HQ
Fruits	100–300 g	240 g	240 g	290 g	290 g
Legumes *	0–100 g	120 g	78 g	96.5 g	72.5 g
Nuts and seeds	0–75 g	45 g	25 g	30 g	30 g
Vegetables	200–600 g	497.5 g	497.5 g	497 g	497 g
Whole grains *	232 g	230 g	230 g	233 g	229 g

LQ = Low-quality day menu, HQ = High-quality day menu. * Dry, raw grain.

**Table 6:** Summary of PDCAAS results and judged protein quality.

	PDCAAS	Increase in PQ	Judged Quality	1st Limiting IAA	2nd Lim IAA
1 LQ	88	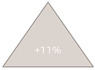	Good	Val	89 (Lys)
1 HQ	98		Good	Lys, Val	106 (Leu)
2 LQ	74	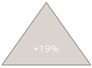	Low	Lys	106 (Val)
2 HQ	88		Good	Lys	109 (SAA)

PDCAAS = Protein Digestibility-Corrected Amino Acid Score, PQ = Protein quality, IAA = Indispensable Amino Acid, Lys = lysine, SAA = Sulphur amino acids, Val = valine, Leu = leucine.

**Table 7:** Summary of DIAAS results and judged protein quality.

	DIAAS	Increase in PQ	Judged Quality	1st Limiting AA	2nd Lim AA
1 LQ	76	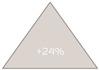	Good	Lys	84 (Val)
1 HQ	94		Good	Lys	98 (Val)
2 LQ	71	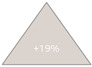	Low	Lys	101 (Val)
2 HQ	83		Good	Lys	101 (SAA)

DIAAS = Digestible Indispensable Amino Acid Score, PQ = Protein quality, IAA = Indispensable Amino Acid, Lys = lysine, SAA = Sulphur amino acids, Val = valine.

## Data Availability

The data presented in this study are available on request from the corresponding author.

## Supplementary Materials

The following supporting information can be downloaded at: www.mdpi.com/article/10.3390/nu14051088/s1, Table S1: Amino acid and protein content, true protein digestibility and ileal indispensable amino acid digestibility for each food ingredient; Table S2: Average values of true protein digestibility of some food items.
