# Supplementary material for: Protein Quality Changes of Vegan Day Menus with Different Plant Protein Source Compositions
Source: Nutrients. 2022 Mar 4;14(5):1088. doi: 10.3390/nu14051088 (PMC8912849; doi:10.3390/nu14051088)
Supplement: Supplementary file 1 [file nutrients-14-01088-s001.zip › Supplementary materials references.pdf]

References Supplementary information:

1. Murlin, J.R.; Marshall, M.E.; Kochakian, C.D. Digestibility and Biological Value of Whole Wheat Breads as Compared with White Bread. J. Nutr. 1941, 22, 573–588, https://doi.org/10.1093/jn/22.6.573.
2. Food and Agriculture Organization of the United Nations. Protein Quality Evaluation: Report of the Joint FAO/WHO Expert Consultation-Bethesda, MD 4-8 December 1989. Food and Agriculture Organization of the United Nations, 1991.
3. Processing Effects on Some Antinutritional Factors and In vitro Multienzyme Protein Digestibility (IVPD) of Three Tropical Seeds: Breadnut (Artocarpus altilis), Cashewnut (Anacardium occidentale) and Fluted Pumpkin (Telfairia occidentalis). Pak. J. Nutr. 2005, 4, 250–256, https://doi.org/10.3923/pjn.2005.250.256.
4. Jørgensen, H.; Brandt, K.; Lauridsen, C. Year rather than farming system influences protein utilization and energy value of vegetables when measured in a rat model. Nutr. Res. 2008, 28, 866–878, https://doi.org/10.1016/j.nutres.2008.09.012.
5. Boye, J.; Wijesinha-Bettoni, R.; Burlingame, B. Protein quality evaluation twenty years after the introduction of the protein digestibility corrected amino acid score method. Br. J. Nutr. 2012, 108, S183–S211, https://doi.org/10.1017/s0007114512002309.
6. Rutherfurd, S.M.; Fanning, A.C.; Miller, B.J.; Moughan, P.J. Protein Digestibility-Corrected Amino Acid Scores and Digestible Indispensable Amino Acid Scores Differentially Describe Protein Quality in Growing Male Rats. J. Nutr. 2015, 145, 372–379, https://doi.org/10.3945/jn.114.195438.
7. Nosworthy, M.G.; Franczyk, A.J.; Medina, G.; Neufeld, J.; Appah, P.; Utioh, A.; Frohlich, P.; House, J.D. Effect of Processing on the in Vitro and in Vivo Protein Quality of Yellow and Green Split Peas (Pisum sativum). J. Agric. Food Chem. 2017, 65, 7790–7796, https://doi.org/10.1021/acs.jafc.7b03597.
8. Martínez-Velasco, A.; Alvarez-Ramirez, J.; Rodríguez-Huezo, E.; Meraz-Rodríguez, M.; Vernon-Carter, E.; Lobato-Calleros, C. Effect of the preparation method and storage time on the in vitro protein digestibility of maize tortillas. J. Cereal Sci. 2018, 84, 7–12, https://doi.org/10.1016/j.jcs.2018.09.016.
9. Nosworthy, M.G.; Medina, G.; Franczyk, A.J.; Neufeld, J.; Appah, P.; Utioh, A.; Frohlich, P.; House, J.D. Effect of processing on the in vitro and in vivo protein quality of red and green lentils (Lens culinaris). Food Chem. 2018, 240, 588–593, https://doi.org/10.1016/j.foodchem.2017.07.129.
10. Nosworthy, M.G.; Medina, G.; Franczyk, A.J.; Neufeld, J.; Appah, P.; Utioh, A.; Frohlich, P.; House, J.D. Effect of Processing on the In Vitro and In Vivo Protein Quality of Beans (Phaseolus vulgaris and Vicia Faba). Nutrients 2018, 10, 671, https://doi.org/10.3390/nu10060671.
11. Ciuris, C.; Lynch, H.M.; Wharton, C.; Johnston, C.S. A Comparison of Dietary Protein Digestibility, Based on DIAAS Scoring, in Vegetarian and Non-Vegetarian Athletes. Nutrients 2019, 11, 3016, https://doi.org/10.3390/nu11123016.
12. Dina, A.; El-Chaghaby, G. NUTRITIONAL QUALITY, AMINO ACID PROFILES, PROTEIN DIGESTIBILITY CORRECTED AMINO ACID SCORES AND ANTIOXIDANT PROPERTIES OF FRIED TOFU AND SEITAN. 2019, 176–190.
13. Kashyap, S.; Varkey, A.; Shivakumar, N.; Devi, S.; H, R.R.B.; Thomas, T.; Preston, T.; Sreeman, S.; Kurpad, A.V. True ileal digestibility of legumes determined by dual-isotope tracer method in Indian adults.. Am. J. Clin. Nutr. 2019, 110, 873–882, https://doi.org/10.1093/ajcn/nqz159.

14. González, A.; Cruz, M.; Losoya, C.; Nobre, C.; Loredo, A.; Rodríguez, R.; Contreras, J.; Belmares, R. Edible mushrooms as a novel protein source for functional foods. Food Funct. 2020, 11, 7400–7414, https://doi.org/10.1039/d0fo01746a.
15. Gomes Almeida Sá, C.B.; Franco, M.Y,M.; Mattar, B.A.C. Food processing for the improvement of plant proteins digestibility. Crit. Rev. Food Sci. Nutr. 2020, 60, 3367–3386, doi:10.1080/10408398.2019.1688249.
16. Reynaud, Y.; Buffière, C.; Cohade, B.; Vauris, M.; Liebermann, K.; Hafnaoui, N.; Lopez, M.; Souchon, I.; Dupont, D.; Rémond, D. True ileal amino acid digestibility and digestible indispensable amino acid scores (DIAASs) of plant-based protein foods. Food Chem. 2020, 338, 128020, https://doi.org/10.1016/j.foodchem.2020.128020.
17. USDA, U.S.D.O.A. FoodData Central. FoodData Central.
